# Internal and External Loads during Formal Training and Competition, Physical Capacities, and Technical Skills in Youth Basketball: A Comparison between Starters and Rotation Players

**DOI:** 10.5114/jhk/201159

**Published:** 2025-04-30

**Authors:** Gonçalo Gonçalves, Paulo Neta, João Ribeiro, Eduardo Guimarães

**Affiliations:** 1 Centre of Research, Education, Innovation and Intervention in Sport (CIFI2D), Faculty of Sport, University of Porto, Porto, Portugal.; 2 Department of Physical Education and Sports Sciences, University of Maia, Maia, Portugal.

**Keywords:** training and game demands, physical performance, basketball skills, playing role, youth players

## Abstract

This preliminary study had two aims: (i) to compare the internal and external loads between training sessions and official games in youth basketball players; (ii) to investigate the effects of the playing role on physical capacities, basketball skills, and internal and external loads accumulated during training and competition. Thirteen under-16 male basketball players, aged 15.30 ± 0.88 years, were followed over 10 consecutive weeks and assigned into two groups according to their playing role: starters and rotation players. Prior to the 10-week monitoring period, players’ training experience, basic anthropometrics, physical capacities, and basketball skills were assessed. Then, internal and external loads were monitored in formal training and official competition. Data were analyzed using non-parametric techniques. Players attained significantly higher values in most measures of internal and external loads during official games (r = 0.82 to 0.88, p < 0.01). Starters outperformed rotation players in the 20-m sprint (r = 0.61, p < 0.05), the lane agility drill (r = 0.77, p < 0.01), and the control dribble (r = 0.73, p < 0.01). They also reached a lower HR_peak_ in practice (r = 0.65, p < 0.05), but a higher %HR_peak_ (r = –0.61, p < 0.05) and greater scores in the summated-heart-rate-zones model (r = –0.61, p < 0.05) as well as the dynamic stress load during official games (r = –0.57, p < 0.05). The findings highlight that young basketball players apparently do not train as intensely as they play. Moreover, it was during competition that starting players experienced higher internal and external loads. It is recommended that basketball coaches regularly monitor loads in both formal training and competition to closely match game demands when planning their practices. Finally, it is suggested that future research includes larger sample sizes to provide a more comprehensive understanding of both internal and external loads in youth basketball.

## Introduction

As players grow and develop throughout their sporting careers, the sport they play naturally becomes more challenging and demanding (Lloyd and Oliver, 2012). In basketball, the game turns faster, more physical, and even more intense with an increasing competitive level (Guimarães et al., 2021). Also, modern basketball is widely characterized as a highly intermittent sport in which game actions change approximately every 2 s (Ben Abdelkrim et al., 2007). Hence, striving to consistently optimize high level performances towards excellence in adulthood, youth basketball coaches often pose a seemingly timeless question: do players and teams practice as intensely as they play?

To properly prepare players to excel in both training and competition, it is of utmost importance that coaches monitor their internal and external loads (Russell et al., 2021; Scanlan et al., 2014). Internal load refers to players’ physiological responses to stimuli induced by training or competition, and is commonly assessed using heart rate (HR) responses [e.g., maximum HR (HR_peak_), mean HR (HR_mean_), and relative maximum heart rate (%HR_peak_)], HR derived measures [e.g., summated-heart-rate-zones (SHRZ) model (Edwards, 1993)], perceptual indicators of exercise intensity [e.g., session rating of perceived exertion (sRPE)], and indirect markers of metabolic stress (e.g., blood lactate concentration) (Halson, 2014). In turn, external load represents a quantification of physical stimuli and movement patterns performed by the players during training or competition, independently of their internal response (Espasa-Labrador et al., 2023). Mostly obtained using time motion analysis (e.g., notational video, software assisted video, inertial measurement units, or local/global positioning systems), external load generally comprises distances, speeds, jumps, steps, accelerations, as well as metrics of effort (e.g., player load) and fatigue [e.g., dynamic stress load (DSL)] (Halson, 2014).

It is acknowledged that a joint monitoring of internal and external load provides coaches with objective data leading to an encompassing control of training demands (Scanlan et al., 2014). This allows them to design more effective practice regimes, detached from the risk of overtraining and severe injury, as well as ensure sustainable long-term development (Halson, 2014). In addition, the data gathered across the season enable coaches to create representative tasks that simulate key aspects of performance aiming to promote adaptations and a positive transfer of training stimuli to competition (Sosa-Martin et al., 2024).

Although loads accumulated during training and competition are a common matter of debate among coaches, the available data from youth basketball players remain limited. Also, the few existing published reports vary in terms of sampling, study duration, and procedures used to monitor training loads. For example, Lupo et al. (2017) and Arede et al. (2022) quantified internal load exclusively during training sessions, whereas Alonso et al. (2020, 2022) monitored external load solely in official games. In contrast, Brandão et al. (2019) and Arede et al. (2020) examined the two types of loads during both training and competition. However, the former did it for a short period of time (4 practices and 1 game within a single week), while the latter used a subjective measure to quantify internal load in 19 training sessions and three friendly matches. Given that such discrepancies among reports may produce inconsistent findings, there still exists a strong call for coaches to better understand whether youth basketball players and teams, in fact, practice as intensely as they play.

Furthermore, the complex nature of basketball also requires that coaches prepare their players to display remarkable levels of physical capacities and technical skills from an early age (Mačinskas et al., 2023; Matulaitis et al., 2019; Stojanović et al., 2018). In fact, the players’ ability to perform intense activities such as sprinting, jumping, shuffling, and changing direction combined with their proficiency in shooting, passing, and dribbling the ball have been strongly linked to individual and team success in youth basketball (Guimarães et al., 2019). Knowing that the first minutes of a game are the most physically demanding (Ben Abdelkrim et al., 2007), coaches should carefully select their starting five. While starters tend to outperform their non-starter peers in certain physical tests (Arede et al., 2020), this appears to have little impact on the internal and external load experienced by players of different playing statuses (Arede et al., 2020; Sansone et al., 2023). Nevertheless, the limited body of research on youth basketball highlights the need for further investigation into this topic.

Therefore, the purpose of the present study was two-fold: (i) to compare the internal and external loads between training sessions and official games in under-16 male basketball players, and (ii) to investigate the effects of the playing role (i.e., starters vs. rotation players) on physical capacity, basketball skills, and internal and external loads accumulated during training sessions and official games. It was hypothesized that internal and external loads would be greater during competition than in formal training. It was also expected that starting players would outperform their peers in physical capacities and basketball skills, as well as show lower internal and external loads during both training sessions and official games.

## Methods

### Participants

Thirteen adolescent male basketball players (age = 15.30 ± 0.88 years; training experience = 7.31 ± 2.53 years; body height = 174.33 ± 7.49 cm; body mass = 65.49 ± 9.24 kg) coming from an under-16 team were recruited to participate in this study. The players, who competed in the first division of the U16 Regional Championship and qualified to play the U16 National Championship, were assigned into two groups according to their role in the 10 official games: starters (players who started in ≥70% of games and averaged ≥28 min per game; *n* = 5) and rotation players (remaining players who mostly came off the bench, averaging ≤12 min per game; *n* = 8). Written informed consent was obtained from parents or legal guardians, as well as individual assent from each player. The study was approved by the Ethics Committee of the Faculty of Sport, University of Porto, Porto, Portugal (protocol code: CEFADE 35 2022; approval date: 18 January 2023), and the club gave formal permission for data collection.

### Design and Procedures

A longitudinal study design was employed to quantify the internal and external loads accumulated by a restricted number of youth basketball players across time. Players were monitored over 10 consecutive in-season weeks, and loads were compared between formal training and competition. Altogether, individual internal and external load measures were assessed during 32 basketball training sessions and 10 official games (5 home and 5 away games). Across the 10-week data collection period, a total of 316 and 95 observations were obtained from training sessions and official games, respectively. Furthermore, players were divided into two groups to examine the differences between starters and rotation players not only in internal and external loads during training sessions and official games, but also in physical capacities and basketball skills assessed prior to the monitoring period. All assessments occurred from November 2022 through to January 2023, and a typical training week consisted of four practices (rest day on Wednesdays) with a single game on the weekend. Training sessions took place in two different courts in the late afternoon or evening, had duration of 83.37 ± 8.84 min, and were fully prescribed by the team’s head coach without any intervention by the research team. A typical practice was composed of individual technique exercises, followed by small-sided games with numerical advantages, shooting drills, and formal 5x5 games.

### Measures

Prior to the 10-week monitoring period, players’ training experience, basic anthropometrics, physical capacities, and basketball skills were assessed. Training experience, expressed as accumulated years of formal basketball training, was obtained from registration histories available on the official website of the Portuguese Basketball Federation. Body height was measured without shoes and with the player’s head positioned in the Frankfurt plane using a Harpenden stadiometer (Holtain Ltd., Crymych, UK) with precision of 0.1 cm. Body mass was obtained using a bio-impedance scale (Tanita®BC-418MA, Tanita Corp., Tokyo, Japan) with precision of 100 g. All measurements were taken by experienced anthropometrists following the International Working Group on Kinanthropometry protocols (Ross and Marfell-Jones, 1995). From the set of physical and skill tests employed, it is worth mentioning that the Yo-Yo Intermittent Recovery Test - Level 1 (Yo-Yo IR1) was used not only to evaluate high-intensity aerobic capacity, but also to determine individual HR_peak_. As done in several previous studies involving samples of basketball players (Fox et al., 2018; Scanlan et al., 2014), this individual HR_peak_ reference value was used exclusively for SHRZ calculations and was replaced only if a player attained a higher HR_peak_ during formal training or competition (Berkelmans et al., 2018).

Before every training session and official game, each player was carefully equipped with an elastic strap holding a Garmin HRM-Dual^™^ HR monitor (Garmin Inc., Kansas, USA) and a custom vest containing a WIMU PRO™ device (RealTrack Systems, Almería, Spain). To avoid inter-unit error, each player wore the same HR monitor and WIMU PRO™ device in every practice and game, as recommended by Gaudino et al. (2014).

At the end of each basketball practice and game, all data collected were exported and analyzed using the SPRO software and its custom algorithm (RealTrack Systems, Almería, Spain). Then, the data were trimmed so that the active time of each player was defined as follows: (i) training sessions: total practice time excluding breaks between tasks and bench time in formal 5x5 game; (ii) official games: total game time when the player was on the court, including all stoppages except time outs and quarter/half breaks (Russell et al., 2021). Warm-up and cool down activities were not considered neither in training nor in competition.

### Internal Load

Internal load was measured during training sessions and official games using Garmin HRM-Dual^™^ HR monitors (Garmin Inc., Kansas, USA), which transmit real-time HR data to the WIMU PRO^™^ system over ANT+® technology with a sampling frequency of 4 Hz, a process previously validated with high reliability and precision (Molina-Carmona et al., 2018). The following internal load measures were assessed: (i) HR_peak_: greatest number of bpm reached in training sessions and official games; (ii) HR_mean_: average number of bpm recorded in training sessions and official games; (iii) %HR_peak_: percentage of the maximum HR obtained in training sessions and official games; (iv) percentage of time spent in 50–60% HR_peak_ (zone 1), 60–70% HR_peak_ (zone 2), 70–80% HR_peak_ (zone 3), 80–90% HR_peak_ (zone 4), and 90–100% HR_peak_ (zone 5): duration in five different HR zones in training sessions and official games. Then, an objective measure of internal load during training sessions and official games was calculated using Edwards’s (1993) SHRZ model. The equation, frequently reported in recent literature to quantify internal load in basketball players (Fox et al., 2018; Scanlan et al., 2014), uses a specific formula that multiplies weighted HR zones by the time spent in each of the five aforementioned zones (Equation 1):

***Equation*** 1:


$$\begin{array}{l} SHRZ=(duration\;\;in\;\;zone\;1\;\times \;1)+(duration\;\;in\;\;zone\;2\;\times \;2)+\;(duration\;\;in\;\;zone\;3\;\times \;3)\\ +\;(duration\;\;in\;\;zone\;4\;\times \;4)\;+\;\;(duration\;\;in\;\;zone\;5\;\times \;5) \end{array}$$

To control for differences in active time among players, the percentage of time spent in five HR_peak_ zones and SHRZ were calculated per minute.

### External Load

External load was monitored during training sessions and official games using the WIMU PRO™ (RealTrack Systems, Almería, Spain). This inertial device contains four 3-axis accelerometers, three 3-axis gyroscopes, and a 3-axis magnetometer, which measure and detect movement through a micro-electromechanical system, offering an adjustable sampling frequency ranging from 10 to 1000 Hz (Gómez-Carmona et al., 2019). The system showed adequate reliability to measure specific team sport movements (Bastida-Castillo et al., 2018) and has been frequently used to evaluate physical demands in youth basketball players (Arede et al., 2020; Vásquez-Guerrero et al., 2019). It should be noted, however, that the LPS antenna system was not employed in this study; therefore, the findings are based solely on data from the inertial measurement units. In any case, the following external load measures were assessed: (i) player load: vector sum of device accelerations in the three orthogonal axes (vertical, anteroposterior, and lateral) calculated from Equation 2 (Reche-Soto et al., 2019); (ii) DSL: total weighted impacts, based on accelerometer values over 2 g (Reche-Soto et al., 2022); (iii) impacts: the number of impacts when the body experienced a g-force greater than 5 g (Puente et al., 2017); (iv) steps: the number of body movements with a flight time less than 400 ms (Román et al., 2019); (v) jumps: the number of body movements with a flight time higher than 400 ms (Pino-Ortega et al., 2018). To control for differences in active time among players, all external load variables were calculated per minute.

***Equation* 2:**


$$\begin{array}{l} PL_{n}=\sqrt{\frac{{\left(X_{n}-X_{n-1}\right)}^{2}+{\left(Y_{n}-Y_{n-1}\right)}^{2}+{\left(Z_{n}-Z_{n-1}\right)}^{2}}{100}}\\ Accumulated\;PL=\sum_{n=0}^{m}PL_{n}\times 0.01 \end{array}$$

### Physical Capacities

Physical capacities were assessed using the following four standardized field-based tests: (i) 20-m sprint: players stood on the 0 m mark and, when ready, ran in a straight line at full speed (Kirkendall et al., 1987). Time (s) was recorded using the photoelectric cells system Speed Trap II (Brower Timing Systems LLC., Draper, UT, USA); (ii) lane agility drill: players had to run forward, side shuffle to the right, run backward, side shuffle to the left, and repeat the same rectangle-shape course in the opposite direction as fast as possible around the lines limiting the basketball restricted area (Stojanović et al., 2019). Time (s) was recorded using the photoelectric cells system Speed Trap II (Brower Timing Systems LLC., Draper, UT, USA); (iii) seated medicine ball throw: players threw a 3-kg ball straight forward as far as possible while seated on the floor with their legs fully stretched and their backs against a wall (Mayhew et al., 1997). The distance (m) was recorded using a tape measure; (iv) countermovement jump: players performed this type of the vertical jump as indicated by Bosco et al. (1983). Jumping height (cm) was measured using an Ergo Tester (Globus, Codognè, Italy); (v) the Yo-Yo IR1: players performed repeated 40-m (2×20-m) runs with a 10-s active recovery period in between (Bangsbo et al., 2008). The total distance covered (m) was recorded.

### Basketball Skills

Basketball skills were assessed using three out of the four field-based tests included in the battery developed by the American Alliance for Health, Physical Education, Recreation and Dance (Hopkins et al., 1984): (i) speed shooting: players shot the ball from five positions (0º, 45º and 90º), collected their own rebound, dribbled to another designated position, and repeated this sequence as quickly as possible over 60 s. The total number of points obtained was recorded; (ii) passing: players performed chest passes against six targets marked on a wall and retrieved the ball while moving laterally over 30 s. The total number of points obtained was recorded; (iii) control dribble: players dribbled the ball while running as quickly as possible in an obstacle course defined by six cones. Time (s) was recorded using the photoelectric cells system Speed Trap II (Brower Timing Systems LLC., Draper, UT, USA).

### Statistical Analysis

Descriptive statistics are presented as means and standard deviations (Mean ± SD). The Shapiro-Wilk test was used for normality checks, and no significant deviations were found. However, given the sample size, the data were analyzed using non-parametric techniques. The Wilcoxon signed-rank test was used to determine differences between training sessions and official games in internal and external loads, whereas the Mann-Whitney *U* test was used to compare groups in physical capacities, basketball skills, as well as internal and external loads during practice and competition. Furthermore, the value of the correlation coefficient *r* was computed as a measure of effect size and interpreted as follows: 0.10 (small effect size), 0.30 (medium effect size), and 0.50 (large effect size) (Cohen, 1992). All data analyses were performed using the IBM SPSS 29.0 (IBM Corp., Armonk, NY, USA), and the alpha level was set at 0.05.

## Results

**Figure 1:**
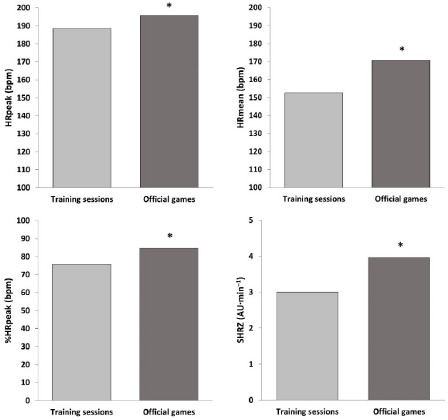
Internal load measures during training sessions and official games (* p < 0.01). HR_peak_ = maximum heart rate; HR_mean_ = mean heart rate; %HR_peak_ = percentage of maximum heart rate; SHRZ = summated-heart-rate-zones model; AU = arbitrary units

**Figure 2:**
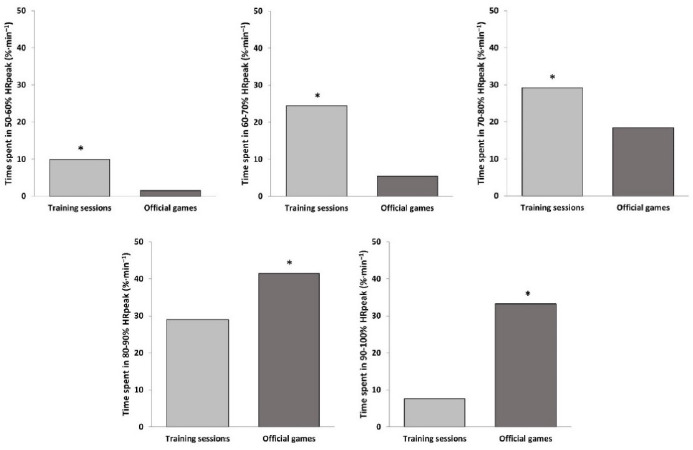
Percentage of time spent in five different heart rate zones during training sessions and official games (* *p* < 0.01). HR_peak_ = maximum heart rate

**Figure 3:**
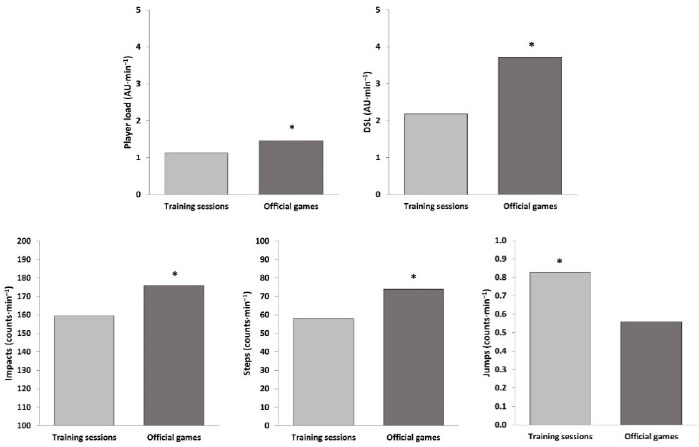
External load measures during training sessions and official games (* *p* < 0.01). DSL = dynamic stress load; AU = arbitrary units

The descriptive statistics for internal and external load outcomes during training sessions and official games are shown in Table 1. Youth basketball players attained a significantly higher HR_peak_ (Z = 2.97; *p* < 0.01; *r =* 0.82), HR_mean_ (Z = 3.18; *p* < 0.01; *r =* 0.88), %HR_peak_ (Z = 3.18; *p* < 0.01; *r =* 0.88), and SHRZ (Z = 3.18; *p* < 0.01; *r =* 0.88) during competition (Figure 1). Also, players spent more time during training sessions between 50 and 80% HR_peak_ (Z = –3.18; *p* < 0.01; *r =* –0.88), while it was during games that they spent longer time between 80 and 100% HR_peak_ (Z = 3.18; *p* < 0.01; *r =* 0.88) (Figure 2). Apart from the number of jumps, basketball players reached significantly greater player load (Z = 3.18; *p* < 0.01; *r =* 0.88) and DSL (Z = 3.11; *p* < 0.01; *r =* 0.86), as well as performed more impacts (Z = 3.18; *p* < 0.01; *r =* 0.88) and steps (Z = 3.18; *p* < 0.01; *r =* 0.88) in competition than in formal training (Figure 3).

**Table 1:** Descriptive statistics for internal and external load during training sessions and official games [Wilcoxon signed-rank test (*Z*) and correlation coefficient (*r*)].

Variables	Training sessions	Official games	*Z* (*r*)
	Mean ± SD	Mean ± SD	
*Internal load*			
HR_peak_ (bpm)	188.55 ± 7.98	195.66 ± 7.15	2.97 (0.82)*
HR_mean_ (bpm)	152.64 ± 7.76	170.75 ± 6.88	3.18 (0.88)*
%HR_peak_ (bpm)	75.84 ± 2.38	84.88 ± 2.37	3.18 (0.88)*
Time spent in 50–60% HR_peak_ (%•min^–1^)	9.86 ± 3.53	1.56 ± 2.14	–3.18 (–0.88)*
Time spent in 60–70% HR_peak_ (%•min^–1^)	24.41 ± 6.07	5.44 ± 2.40	–3.18 (–0.88)*
Time spent in 70–80% HR_peak_ (%•min^–1^)	29.15 ± 3.43	18.35 ± 4.41	–3.18 (–0.88)*
Time spent in 80–90% HR_peak_ (%•min^–1^)	28.97 ± 7.25	41.42 ± 3.82	3.18 (0.88)*
Time spent in 90–100% HR_peak_ (%•min^–1^)	7.61 ± 3.45	33.22 ± 5.18	3.18 (0.88)*
SHRZ (AU•min^–1^)	3.00 ± 0.24	3.96 ± 0.25	3.18 (0.88)*
*External load*			
Player load (AU•min^–1^)	1.12 ± 0.06	1.46 ± 0.11	3.18 (0.88)*
DSL (AU•min^–1^)	2.18 ± 0.66	3.72 ± 1.50	3.11 (0.86)*
Impacts (counts•min^–1^)	159.54 ± 9.75	175.92 ± 13.66	3.18 (0.88)*
Steps (counts•min^–1^)	57.84 ± 4.58	73.92 ± 8.93	3.18 (0.88)*
Jumps (counts•min^–1^)	0.83 ± 0.23	0.56 ± 0.15	–3.18 (–0.88)*

HR_peak_ = maximum heart rate; HR_mean_ = mean heart rate; %HR_peak_ = percentage of maximum heart rate; SHRZ = summated-heart-rate-zones model; DSL = dynamic stress load; AU = arbitrary units; * p < 0.01

Table 2 shows the comparisons between starters and rotation players for physical capacity and basketball skill field-based tests. Starters only outperformed their peers in the 20-m sprint test (Z = 2.21; *p* < 0.05; *r =* 0.61) and the lane agility drill (Z = 2.78; *p* < 0.01; *r =* 0.77). In addition, starters were significantly more skilled only in the control dribble test (Z = 2.63; *p* < 0.01; *r =* 0.73).

**Table 2:** Descriptive statistics for physical capacities and basketball skills of starting and rotation young basketball players [Mann-Whitney U test (*Z*) and correlation coefficient (*r*)].

Variables	Starters	Rotation	*Z* (*r*)
	Mean ± SD	Mean ± SD	
*Physical capacities*			
20-m sprint (s)	3.17 ± 0.06	3.36 ± 0.20	2.21 (0.61)*
Lane agility drill (s)	12.04 ± 0.37	13.30 ± 0.64	2.78 (0.77)**
Seated medicine ball throw (m)	4.87 ± 0.23	4.51 ± 0.45	–1.46 (–0.41)
Countermovement jump (cm)	43.26 ± 2.11	38.70 ± 4.04	–1.91 (–0.53)
Yo-Yo IR1 (m)	1488.00 ± 476.15	1420.00 ± 489.43	–0.51 (–0.14)
*Basketball skills*			
Speed shot shooting (points)	39.60 ± 4.56	34.50 ± 4.84	–1.76 (–0.49)
Passing (points)	108.00 ± 9.19	107.25 ± 16.56	0.29 (0.08)
Control dribble (s)	16.40 ± 0.27	17.47 ± 0.85	2.63 (0.73)**

Yo-Yo IR1 = Yo-Yo Intermittent Recovery Test - Level 1; * p < 0.05; ** p < 0.01

The comparisons between starters and rotation players for internal and external loads in training sessions and official games are presented in Table 3. During practice, significant differences were only found in the HR_peak_ in favor of those players who frequently came off the bench (Z = 2.34; *p*<0.05; *r =* 0.65). In contrast, starters showed higher values of %HR_peak_ (Z = –2.20; *p* < 0.05; *r =* –0.61), SHRZ (Z= –2.20; *p* < 0.05; *r =* –0.61), and DSL (Z = –2.05; *p* < 0.05; *r =* –0.57) during formal competition.

**Table 3:** Descriptive statistics for internal and external load during training sessions and official games of starting and rotation young basketball players [Mann-Whitney U test (*Z*) and correlation coefficient (*r*)].

		Training sessions	
Variables	Starters	Rotation	*Z* (*r*)
	Mean ± SD	Mean ± SD	
*Internal load*			
HR_peak_ (bpm)	182.83 ± 3.76	192.13 ± 7.95	2.34 (0.65)*
HR_mean_ (bpm)	148.03 ± 4.98	155.52 ± 8.03	1.61 (0.45)
%HR_peak_ (bpm)	74.83 ± 2.11	76.48 ± 2.44	0.88 (0.24)
Time spent in 50–60% HR_peak_ (%•min^–1^)	10.21 ± 3.81	9.64 ± 3.60	0.15 (0.04)
Time spent in 60–70% HR_peak_ (%•min^–1^)	26.11 ± 5.82	23.34 ± 6.36	–0.59 (–0.16)
Time spent in 70–80% HR_peak_ (%•min^–1^)	30.84 ± 2.58	28.10 ± 3.62	–1.61 (–0.45)
Time spent in 80–90% HR_peak_ (%•min^–1^)	27.21 ± 6.80	30.08 ± 7.75	0.73 (0.20)
Time spent in 90–100% HR_peak_ (%•min^–1^)	5.63 ± 3.60	8.85 ± 2.92	1.90 (0.53)
SHRZ (AU•min^–1^)	2.91 ± 0.22	3.06 ± 0.25	0.88 (0.24)
*External load*			
Player load (AU•min^–1^)	1.15 ± 0.03	1.10 ± 0.07	–1.61 (–0.45)
DSL (AU•min^–1^)	2.56 ± 0.74	1.93 ± 0.50	–1.76 (–0.49)
Impacts (counts•min^–1^)	156.73 ± 8.82	161.30 ± 10.45	0.88 (0.24)
Steps (counts•min^–1^)	58.34 ± 2.78	57.53 ± 5.58	–0.59 (–0.16)
Jumps (counts•min^–1^)	0.79 ± 0.17	0.85 ± 0.27	0.44 (0.12)
		**Official games**	
**Variables**	**Starters**	**Rotation**	***Z* (*r*)**
	**Mean ± SD**	**Mean ± SD**	
*Internal load*			
HR_peak_ (bpm)	194.12 ± 1.58	196.62 ± 9.13	0.00 (0.00)
HR_mean_ (bpm)	170.02 ± 2.76	170.58 ± 8.76	–0.15 (–0.04)
%HR_peak_ (bpm)	86.44 ± 1.21	83.91 ± 2.45	–2.20 (–0.61)*
Time spent in 50–60% HR_peak_ (%•min^–1^)	0.50 ± 0.36	2.23 ± 2.54	1.76 (0.49)
Time spent in 60–70% HR_peak_ (%•min^–1^)	4.15 ± 1.91	6.25 ± 2.42	1.76 (0.49)
Time spent in 70–80% HR_peak_ (%•min^–1^)	16.23 ± 4.21	19.68 ± 4.25	1.61 (0.45)
Time spent in 80–90% HR_peak_ (%•min^–1^)	43.33 ± 2.18	40.22 ± 4.24	–1.90 (–0.53)
Time spent in 90–100% HR_peak_ (%•min^–1^)	35.79 ± 6.14	31.62 ± 4.10	–1.46 (–0.41)
SHRZ (AU•min^–1^)	4.13 ± 0.13	3.86 ± 0.25	–2.20 (–0.61)*
*External load*			
Player load (AU•min^–1^)	1.50 ± 0.06	1.43 ± 0.13	–0.44 (–0.12)
DSL (AU•min^–1^)	4.97 ± 1.45	2.93 ± 0.90	–2.05 (–0.57)*
Impacts (counts•min^–1^)	166.47 ± 15.26	181.83 ± 9.11	1.90 (0.53)
Steps (counts•min^–1^)	73.78 ± 4.72	74.00 ± 11.14	0.29 (0.08)
Jumps (counts•min^–1^)	0.60 ± 0.14	0.53 ± 0.17	–0.88 (–0.24)

HR_peak_ = maximum heart rate; HR_mean_ = mean heart rate; %HR_peak_ = percentage of maximum heart rate; SHRZ = summated-heart-rate-zones model; DSL = dynamic stress load; AU = arbitrary units; * p < 0.05

## Discussion

This preliminary study aimed to compare internal and external loads between training sessions and official games in youth basketball while also investigating the effects of the playing role on physical capacities, basketball skills, and the loads accumulated during these activities. Overall findings revealed that players experienced greater internal and external loads in official games compared to training sessions. Also, starters and rotation players presented similar physical and technical performances in most field-based tests. However, starters displayed higher internal and external loads during official games. Although important for the daily practice of coaches, the truth is that, to date, literature on this matter is still scarce. Furthermore, the limited body of knowledge available in youth basketball has produced inconsistent outcomes, likely due to sample specificities and/or distinct methodologies used. This poses problems when comparing the findings. Nevertheless, the discussion was framed around three fundamental questions arising from the study hypotheses.

### Do Youth Basketball Players Practice as Intensely as They Play during Official Games?

Confirming the first hypothesis, the young basketball players broadly attained greater internal and external loads during official games than in training sessions. These findings are in part consistent with previous data from youth basketball. For example, Brandão et al. (2019) reported a higher HR_peak_ in competition compared to formal training, although similar values of SHRZ and total distance traveled were observed in young players from Brazil. It should be noted, however, that discrepancies in study duration might be responsible for these contrasting results, namely in SHRZ. By monitoring players for just one week (four training sessions and one formal game), those authors were apparently not able to observe substantial changes in players’ response to training and competition due to limited variation in practice tasks and game competitiveness. A similar trend to that observed in the present study was shown by Arede et al. (2020) in a sample of basketball players, members of the under-16 Portuguese national team. Although using a subjective measure of internal load, they reported superior mean values of the RPE and slightly higher player load and body impacts in friendly games than during training sessions. The findings are also in agreement with a previous study on elite junior basketball players from Australia, which found a greater HR_mean_ and player load during games compared to 5x5 scrimmage or offensive and defensive drills performed during practice (Montgomery et al., 2010). Official games are so unique in terms of duration, intensity, opposition, motivation, and emotional tension so that only the competition itself seems to properly prepare players to compete. Since throughout the season teams train more often than they play, it is recommended that coaches also incorporate highly demanding 5x5 drills into their training sessions (Montgomery et al., 2010). For better load management, it is suggested that these tasks occur in the middle of the weekly training microcycle, that is, not very close to the previous game, nor the upcoming one.

In turn, the number of jumps per minute was the only measure to show an inverse pattern. The large number of jumps performed by players, for example, during shooting drills may explain this unexpected result. Nonetheless, it appears that training sessions do not meet the internal and external demands required during competition in youth basketball. To reduce this gap, basketball coaches should prioritize training sessions that closely represent the competitive environment. When designing training regimens, it is suggested that coaches (i) minimize inactive time within and between drills, (ii) promote competitiveness wherever possible, (iii) manipulate key task conditions (e.g., field dimensions, numeric relationships, rules) to vary internal and external load according to intended levels of load management, (iv) stimulate players to practice at game speed while carefully controlling the balance between work and rest in order to avoid overtraining, and (v) use available basketball official equipment, namely game and shot clocks, to replicate game-related constraints.

To enhance the quality of practice, it is also important that coaches improve the physical capacities of young players from an early age (Lloyd and Oliver, 2012). For such, coaches must implement not only basketball-specific strength and conditioning workouts, but also technical and tactical tasks involving physical work. Especially in the youth, basketball coaches should not exclusively rely on 5x5 game situations to reproduce official game demands (Torres-Ronda et al., 2016). Instead, they should employ, for example, fast-break activities to enhance running speed, continuous full court drills to develop endurance, or defense exercises to improve agility. This is expected to optimize the long-term development of young players and maximize their potential to achieve success.

### Do Basketball Players Who Most Often Started the Games (i.e., Starters) Outperform Their Peers Who Mostly Came Off the Bench (i.e., Rotation Players) in Physical Capacities and Basketball Skills?

Despite that starters presented better mean values than rotation players in all field-based tests, contrary to the initial expectations, significant differences were only found in the 20-m sprint, the lane agility drill, and the control dribble. Although care must be taken in interpretation due to the small sample size of both groups, the findings do not align with those from Arede et al. (2020) who reported significant differences in favor of starters in the countermovement jump, but similar between-group outcomes in the Yo-Yo IR1 and agility T-test. It is possible that the unique characteristics of each sample may justify these discrepancies. While the present study comprises data coming from a club context, Arede et al. (2020) sampled 12 players, members of the under-16 Portuguese national team who were in their final preparation to compete in the FIBA U16 European Championship, Division B. We, therefore, contend that differences between starters and rotation players are more likely to emerge within a club because in such an environment young players tend to be more different than alike in terms of body size and composition, physical capacity, and skill level, especially when not controlling for the confounding effects of biological maturation and training experience (Guimarães et al., 2019, 2021). On the other hand, regardless of the age-category, national teams commonly show great homogeneity among players. Besides being chosen as the best ones of their age, these players are highly determined to excel in training and competition in order to secure a spot on a restricted roster.

Nevertheless, the present results corroborate previous studies with adult basketball players. For example, Torres-Ronda et al. (2016), Scanlan et al. (2015), and Gonzalez et al. (2012) reported that starters were, on average, more agile than non-starters in men's and women's basketball, respectively. Moreover, Cabarkapa et al. (2023) found similar jumping performance between starting and non-starting male professional players, whereas Scanlan et al. (2021) showed that elite female starters possessed significantly higher aerobic capacity when compared to bench players. Although it was impossible to locate any study contrasting the skill level of starters and rotation players, the present findings suggest that coaches define their starting lineup considering the intrinsic nature of basketball. In a sport where most intense actions are performed at the beginning of the game (Ben Abdelkrim et al., 2007), basketball coaches seem to rely on players who are physically capable of responding better to competitive demands. Knowing that basketball is widely characterized by short and intense bursts of energy as well as dynamic multidirectional movements (Stojanović et al., 2018), the importance of promoting proper athletic development of youth players from an early stage of their sporting careers must be highlighted. This may not only increase opportunities for all team members to start the game and play more minutes, but also maximize players’ potential to achieve either individual or collective success (Guimarães et al., 2019).

### Do Starters Show Lower Internal and External Loads than Rotation Players during Training Sessions and Official Games?

Due to the presumed superior physical performance of starting players, it was expected that they would experience lower internal and external loads during training sessions and official games. These findings, however, did not fully confirm the third hypothesis. In practice, starters attained a significantly lower HR_peak_ than rotation players, but no differences were found in the other measures of internal and external load. Despite contrasting with the initial prediction, these results are somewhat similar to those reported by Arede et al. (2020), that is, no significant differences were observed between starter and non-starter youth players in the RPE (measure of internal load), player load, and body impacts. Similarly, Sansone et al. (2023) reported no significant effects of competition rotation status (starter, rotation, and bench players) on the average or peak external load. Furthermore, Palmer et al. (2021) and Fox et al. (2021) showed equal average session intensity and peak player load, respectively, between starters and bench adult basketball players. During official games, on the other hand, results showed that starters outscored rotation players in %HR_peak_, SHRZ, and DSL. Although these findings are not in line with available data from youth basketball (Arede et al., 2020), they are comparable with a report from professional and semi-professional basketball showing that, in official games, starting players had a greater average intensity than in- and out-rotation bench players (Palmer et al., 2021).

Taken together, these results suggest that youth basketball players' responses to stimuli elicited by training drills are independent of the playing role. Coaches must be aware that the loads implemented by their training regimes are likely to be insufficient to meet game demands, as during competition a distinct picture emerged. The fact that starting players are on the court in the most critical, intense, and decisive moments of the game (e.g., when the score differential is close, the opposition level is higher, and actions have the greatest impact on the final score), makes them experience greater internal and external loads even after controlling for active playing time (Conte et al., 2018; Palmer et al., 2021). In turn, rotation players usually play during “rest periods”, being exposed to lower internal and external game loads (Fox et al., 2022). They should, therefore, be subject to additional loads during training in order to dispose of similar opportunities to participate and succeed in the competition.

Although the present findings extend the reduced available literature on this topic, it is acknowledged that this study is not without limitations. First, it needs to be highlighted that this is a preliminary study; therefore, caution is recommended when generalizing its findings. The second limitation concerns the sample size. Although previous reports have used similar or even smaller samples (Arede et al., 2020; Montgomery et al., 2010), it is acknowledged that using a small number of participants may pose challenges to interpreting and generalizing results. Irrespective of this, it is important to note that the statistical analysis revealed *r*-values of effect size ranging from 0.82 to 0.88 in the Wilcoxon signed-rank test and from 0.57 to 0.77 in the Mann-Whitney *U* test whenever significant differences were observed. This way, the results and conclusions of this study are considered reliable. Third, it is important to note that playing positions were not considered as they were not yet fully established due to the age category of the sampled players. Nevertheless, knowing that basketball coaches are advised to start specializing players for specific positions only at the age of 16 years old (Dezman et al., 2001), it is suggested that future research dealing with older populations examine putative position-specific differences. Fourth, there is no doubt that using the LPS antennas system would provide a higher number of external load variables. However, collecting such data over time across different venues (e.g., several practice gyms, home and away games) would definitely be a difficult task to overcome.

## Conclusions

This study highlights that youth basketball players apparently do not train as intensely as they play. The findings showed that players attained greater internal and external loads in official games than during training sessions. Although challenging, it is recommended that youth basketball coaches regularly monitor loads (preferably using objective measures) in both formal training and competition. Such information is expected to be helpful for coaches to design drills that promote stimuli similar to those encountered during competition, taking into account the complex intertwined relationship between task, individual, and environmental factors. This is of utmost importance to closely match game demands when planning their training sessions, but also for careful load management throughout the training microcycle. Although starters presented similar physical and technical performances in most field-based tests and internal and external loads during training, they experienced higher internal and external loads in official games than rotation players. Through load monitoring, coaches may also gain an in-depth understanding on how training loads vary on an individual scale (the player), in other words, whether internal and external loads observed during most training sessions correspond to the official game demands, for each player, according to their playing position, role, or function within the team. This will undoubtedly improve coaches’ ability to properly design their training regimes. Due to variation between and within youth athletes, the same training will probably not fit all players. Ultimately, it would be helpful for coaches if researchers conducted studies with larger populations capable of providing a more exhaustive understanding of internal and external loads in youth basketball.
